# Reorganization of Brain Functional Connectivity Network and Vision Restoration Following Combined tACS-tDCS Treatment After Occipital Stroke

**DOI:** 10.3389/fneur.2021.729703

**Published:** 2021-10-27

**Authors:** Jiahua Xu, Zheng Wu, Andreas Nürnberger, Bernhard A. Sabel

**Affiliations:** ^1^Institute of Medical Psychology, Medical Faculty, Otto-V.-Guericke University of Magdeburg, Magdeburg, Germany; ^2^Faculty of Computer Science, Otto-V.-Guericke University of Magdeburg, Magdeburg, Germany

**Keywords:** brain networks, vision recovery, stroke, neuron rehabilitation, tACS, tDCS

## Abstract

**Objective:** Non-invasive brain stimulation (NIBS) is already known to improve visual field functions in patients with optic nerve damage and partially restores the organization of brain functional connectivity networks (FCNs). However, because little is known if NIBS is effective also following brain damage, we now studied the correlation between visual field recovery and FCN reorganization in patients with stroke of the central visual pathway.

**Method:** In a controlled, exploratory trial, 24 patients with hemianopia were randomly assigned to one of three brain stimulation groups: transcranial direct current stimulation (tDCS)/transcranial alternating current stimulation (tACS) (ACDC); sham tDCS/tACS (AC); sham tDCS/sham tACS (Sham), which were compared to age-matched controls (*n* = 24). Resting-state electroencephalogram (EEG) was collected at baseline, after 10 days stimulation and at 2 months follow-up. EEG recordings were analyzed for FCN measures using graph theory parameters, and FCN small worldness of the network and long pairwise coherence parameter alterations were then correlated with visual field performance.

**Result:** ACDC enhanced alpha-band FCN strength in the superior occipital lobe of the lesioned hemisphere at follow-up. A negative correlation (*r* = −0.80) was found between the intact visual field size and characteristic path length (CPL) after ACDC with a trend of decreased alpha-band centrality of the intact middle occipital cortex. ACDC also significantly decreased delta band coherence between the lesion and the intact occipital lobe, and coherence was enhanced between occipital and temporal lobe of the intact hemisphere in the low beta band. Responders showed significantly higher strength in the low alpha band at follow-up in the intact lingual and calcarine cortex and in the superior occipital region of the lesioned hemisphere.

**Conclusion:** While ACDC decreases delta band coherence between intact and damaged occipital brain areas indicating inhibition of low-frequency neural oscillations, ACDC increases FCN connectivity between the occipital and temporal lobe in the intact hemisphere. When taken together with the lower global clustering coefficient in responders, these findings suggest that FCN reorganization (here induced by NIBS) is adaptive in stroke. It leads to greater efficiency of neural processing, where the FCN requires fewer connections for visual processing.

## Background

The potential to restore visual fields following central visual system damage has attracted some attention during the last few decades (1–8). Occipital stroke, for example, leads to homonymous hemianopia whereby a quarter or half of the visual field in both eyes is lost following damage (9). This impairs visual functional abilities and quality of life (10), increasing the risk to fall or having difficulties in reading, with secondary deficits such as depression and social isolation (10–14). While visual training can improve visual fields well after the initial spontaneous recovery phase (2, 3, 15), additional recovery of vision can take many months of daily exercises.

To overcome this limitation, efforts were made to use non-invasive brain stimulation (NIBS) as a new therapeutic approach. NIBS was already used for the rehabilitation of different neurological diseases affecting the motor system, memory, language, or cognition (16). NIBS includes different protocols of low-intensity transcranial alternating current stimulation or transcranial direct current stimulation (tACS, tDCS) known to alter brain excitability (17). Especially tDCS was applied to treat different neurological and neuropsychiatric dysfunctions (18, 19). In tDCS, current flows from the anodal to the cathodal electrode, where the anode is thought to enhance (excite) and the cathode reduce (inhibit) neuronal activities (20–22). In contrast, the direction of current flow in tACS alternates between both electrodes and is able to modulate periodic oscillations (17), which can, in turn, entrain endogenous brain oscillation in a frequency- and phase-specific manner (23, 24). With tACS, it is therefore possible to enhance the power, shift the peak, and change the electroencephalogram (EEG) oscillations phase by applying the ACS at a frequency identical or close to those oscillations (25). tACS was already shown to increase parieto-occipital alpha activity and to synchronize cortical oscillations with entrainment of specific frequencies (26), and this impacts the endogenous alpha oscillation with long-lasting “after-effects” (27). When stimulating the brain in the alpha frequency range, for example, this increases alpha power, reflecting neuroplasticity changes rather than entrainment (28). NIBS can also be used to purposely modulate neuron's excitation and inhibition in many neurological diseases with a potential to induce recovery of function (29).

With regard to visual system damage, tACS was shown to enhance recovery following visual cortex or optic nerve damage (30–35). Here, a 10-day treatment course improved visual field size and visual acuity, and it reduced reaction time (RT). The proposed mechanism of action of tACS is that it can modulate synchronization of neuronal network firing of partially damaged “areas of residual vision,” which managed to survive the injury, possibly involving the strengthening of synaptic transmission along the visual pathway and enhancing blood flow. For review, see (36). Indeed, tACS-induced visual improvements significantly correlated with neuronal synchronization changes (5, 34, 37) and enhanced alpha-band activity or power (28, 38).

Concerning tDCS, visual cortex damage leads to hyperactivity of the intact hemisphere, presumably inhibiting the lesioned side (39, 40), and a dual-mode tDCS can reduce visual neglect symptoms (41). That tDCS can improve visual functions was also shown both in normal subjects and in patients with visual system damage. For example, combining tDCS with visual training can improve hemianopic visual fields (42), and in healthy subjects, anodal tDCS of the occipital poles significantly reduces psychophysical surround suppression (43) and enhances occipital blood flow (44). However, little is known about possible frequency-specific neural-plastic mechanisms for vision recovery after occipital stroke, and only few studies explored the potential of NIBS to induce recovery of visual functions in patients suffering from a unilateral occipital stroke (45). Therefore, a better understanding of the neurophysiological mechanism of tACS and tDCS is needed to understand, and eventually maximize, their potential to improve visual fields after occipital stroke.

To learn more about the mechanisms and effects of tACS and tDCS in occipital strokes, we now used both protocols alone or in combination. Specifically, we hypothesized that cathodal tDCS might inhibit the intact visual cortex, reduce its hyperactivity, and thus lower the associated cross-hemispheric inhibition of the damaged visual cortex. Treatment with tACS, on the other hand, might induce endogenous neuronal oscillations on the whole-brain level. Therefore, we now studied both methods alone and in combination. Specifically, we expected that a “double-punch” approach of combined tACS/tDCS would be most effective, because it would simultaneously reduce cross-hemispheric inhibition and enhance excitability of the tissue at or near the lesion site.

To test this hypothesis, we used EEG recordings rather than functional magnetic resonance imaging (fMRI), because the EEG can measure synchronization patterns of the functional connectivity network (FCN) with high (theoretically infinite) time resolution. Indeed, as reported elsewhere, in a similar study, no consistent fMRI-activation changes were observed after NIBS (35). Therefore, the EEG may be the more sensitive and more direct measure of FCN synchronization states and their dynamics. Furthermore, the EEG can detect even physiological alterations independent of energy consumption (46). Here we studied the neurophysiology of brain FCN plasticity in hemianopic stroke patients and describe how FCN reorganization correlates with visual field recovery.

A detailed analysis of visual field recovery was already reported elsewhere (35).

## Materials and Methods

### Demographics

**Figure 1:**
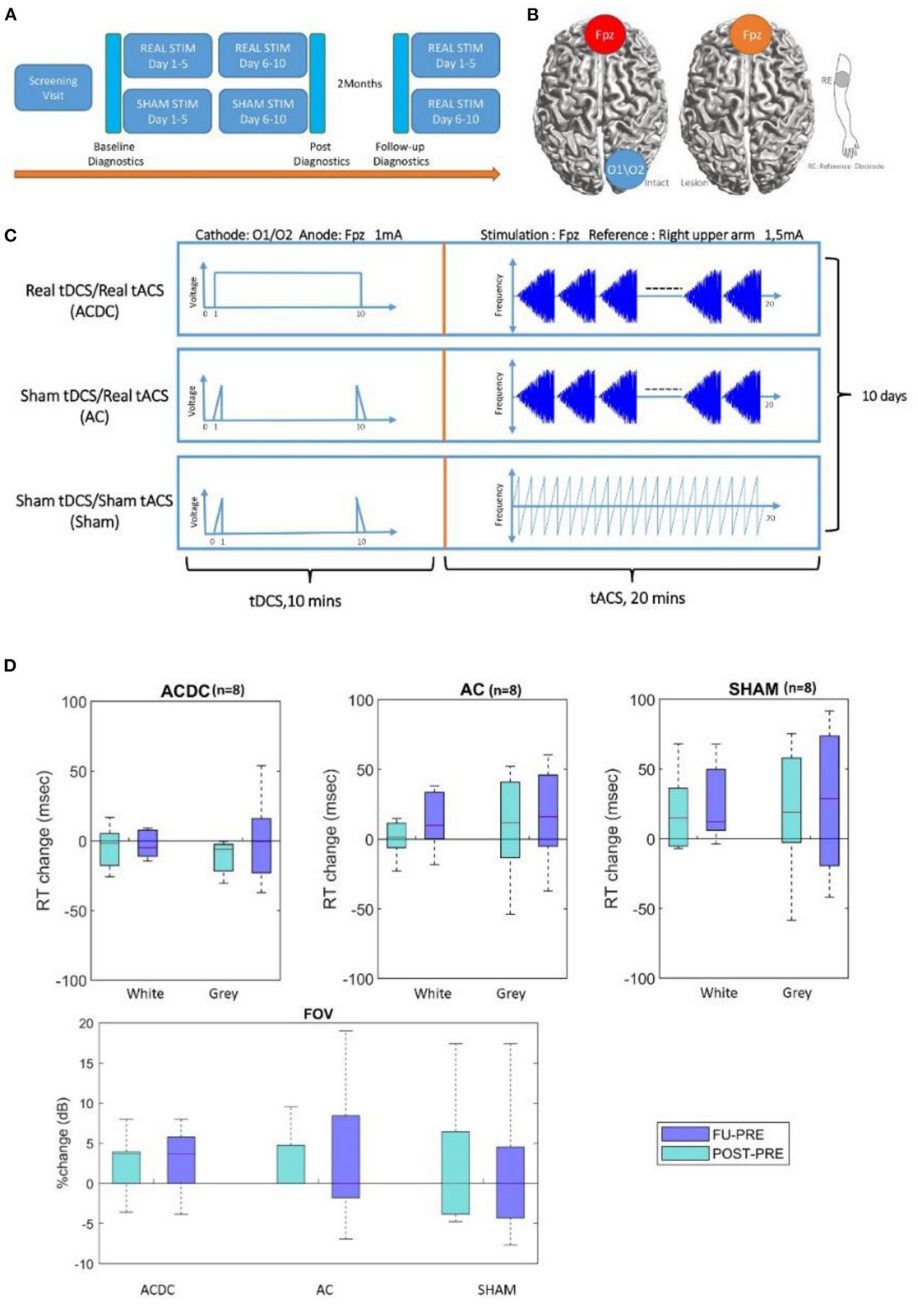
**(A)** The pipeline for experiment time schedule and postdiagnostic, 5 min rsEEG, and visual parameters were recorded before the experiment as a baseline, after 10 days of stimulation, and 2 months later at follow-up. Moreover, all sham patients were offered to receive stimulation treatment after the final evaluation. **(B,C)** Therefore, three treatment groups were compared: tDCS/tACS (ACDC), sham tDCS/tACS (AC), and sham tDCS/sham tACS (SHAM). **(D)** Boxplot of White and gray areas' reaction time in visual field: the percentage change of the reaction time in ACDC decreased while in the two groups it increased. RT, reaction time (unit: millisecond). The bottom part shows the percentage changes in each group, the median of percentage changes of ACDC was positive both in POST and FU. In contrast, AC and SHAM group remains zero. From both high-resolution perimetry and visual field, the ACDC group shows a more promising visual performance improvement than the other two groups. FOV, visual field (unit: dB).

Unilateral occipital stroke patients (*n* = 24) suffering from hemianopia were recruited as previously described (45) and randomly assigned to one of three groups (Figure 1A): tDCS/tACS group (ACDC, *n* = 8, age: mean ± SD = 53.45 ± 14.18), sham tDCS/tACS group (AC, n = 8, age: mean ± SD = 58.25 ± 9.54), and sham tDCS/sham tACS group (Sham, *n* = 8, age: mean ± SD = 63.87 ± 5.38). Their EEG results were compared to 24 healthy subjects (age: mean ± SD = 57.4 ± 10.5) (see Table 1 for details of patients and controls). The study was conducted with the guidelines of the International Conference on Harmonization of Good Clinical Practice and the applicable national legislation in agreement with the Declaration of Helsinki. All participants had signed consent form. The study was approved by the institutional review board of the University Magdeburg. The patient's group identity was known only to the experimenter who performed the stimulation. The participants were informed about their stimulation protocol after completion of follow-up diagnostics at 8 weeks (45).

**Table 1:** Demographics of patients and controls.

**Patients**																					**Controls**	
**ID**	**Group**	**Lesion age (mo)**	**HRP_Pre**			**HRP_Post**			**HRP_FU**			**RT_Pre**		**RT_Post**		**RT_FU**		**Field of vision (dB)**			**Gender**	**Age (y)**
			**Black**	**Gray**	**White**	**Black**	**Gray**	**White**	**Black**	**Gray**	**White**	**Gray**	**White**	**Gray**	**White**	**Gray**	**White**	**Pre**	**Post**	**FU**		
1	2	45	181	29	231	182	28	231	180	33	228	0.50	0.43	0.49	0.43	0.53	0.42	24	25	25	M	24
2	2	61	109	41	291	112	36	293	117	23	301	0.52	0.41	0.52	0.43	0.43	0.42	26	26	25	M	44
3	0	20	60	5	376	56	14	371	55	13	373	0.44	0.35	0.38	0.34	0.42	0.35	26	26	26	M	55
4	0	45	145	65	231	123	92	226	122	85	234	0.47	0.42	0.52	0.46	0.52	0.46	21	20	20	F	75
5	1	19	208	71	162	214	69	158	205	60	176	0.55	0.48	0.55	0.48	0.60	0.48	26	26	26	M	65
6	2	28	125	24	292	115	33	293	110	33	298	0.50	0.39	0.49	0.39	0.50	0.38	28	27	28	M	53
7	1	117	194	8	239	188	16	237	191	23	227	0.49	0.39	0.52	0.39	0.48	0.43	28	28	27	M	61
8	1	29	98	48	295	103	51	287	92	79	270	0.49	0.40	0.48	0.42	0.50	0.43	27	27	27	M	56
9	0	25	149	13	279	150	13	278	149	19	273	0.50	0.38	0.53	0.38	0.46	0.38	27	27	27	M	68
10	1	156	176	19	246	176	15	250	177	16	248	0.51	0.42	0.49	0.41	0.50	0.42	24	27	27	M	51
11	1	21	133	109	199	133	117	191	157	73	211	0.48	0.43	0.53	0.40	0.54	0.41	23	23	24	M	66
12	2	11	198	14	229	198	7	236	193	18	230	0.53	0.48	0.53	0.48	0.53	0.49	27	28	29	M	62
13	0	49	170	79	192	179	39	223	180	56	205	0.45	0.39	0.51	0.41	0.54	0.45	23	27	27	F	48
14	0	27	164	128	149	138	141	162	162	123	156	0.55	0.44	0.54	0.45	0.53	0.45	26	25	25	F	55
15	2	25	186	21	234	176	31	234	177	27	237	0.51	0.37	0.50	0.36	0.51	0.37	28	29	29	F	60
16	2	19	151	34	256	133	45	263	145	32	264	0.60	0.46	0.54	0.43	0.57	0.44	24	24	24	M	50
17	0	12	48	120	273	16	91	334	47	76	318	0.50	0.42	0.50	0.41	0.56	0.43	26	29	26	M	68
18	1	43	199	30	212	202	21	218	200	27	214	0.53	0.36	0.48	0.36	0.50	0.36	21	23	25	F	54
19	2	93	170	16	255	170	6	265	172	4	265	0.52	0.44	0.49	0.38	0.58	0.39	25	27	27	M	67
20	1	6	282	28	131	283	26	132	287	25	129	0.52	0.47	0.56	0.48	0.56	0.49	26	26	26	M	53
21	0	106	194	19	228	191	14	236	193	19	229	0.62	0.53	0.63	0.56	0.63	0.54	22	24	24	M	59
22	1	9	203	12	226	199	10	232	206	9	226	0.55	0.45	0.60	0.45	0.57	0.48	29	29	27	M	57
23	0	7	227	58	156	246	59	136	248	54	139	0.66	0.65	0.74	0.72	0.75	0.72	26	25	24	F	72
24	2	10	159	68	214	177	88	176	160	88	193	0.55	0.42	0.54	0.43	0.54	0.43	27	28	28	M	54
Mean (SD)	0 Sham 1 AC 2 ACDC	40.95 (39.21)	163.70 (51.68)	44.12 (35.92)	233.16 (54.46)	160.83 (57.55)	44.25 (37.25)	235.91 (58.64)	163.54 (54.59)	42.29 (30.98)	235.16 (56.31)	0.51 (0.05)	0.42 (0.06)	0.52 (0.06)	0.42 (0.08)	0.53 (0.07)	0.43 (0.07)	25.41 (2.22)	26.08 (2.18)	25.95 (1.96)	18 M/6 F	57.37 (10.56)

*Gender: M, male; F, female; lesion age: the months since stroke happened; RT, reaction time; Pre, before treatment; Post, after treatment; FU, follow-up; ACDC, rtDCS/rtACS; AC, sham/rtACS; Sham, sham/sham*.

Our patients' hemianopia was caused by ischemic (*n* = 19) or hemorrhagic (*n* = 5) stroke. Their age range was 18–75 years, and lesion age was >6 months. Diagnostic results showed that patients had stable visual field defects across repeated baseline measurements. We found no significant correlation of lesion age with FCN pre–post difference of the two most important parameters (“strength” and “centrality”) on the alpha band, showing that lesion age had no impact on our FCN parameters (see below). It confirms our assumption that network plasticity does not depend on lesion age. In any event, subjects with spontaneous fluctuations and recovery of vision were not entered in the trial. All patients had corrected visual acuity of at least 0.4 (20/50 Snellen) or better. The presence of residual vision and detectable gradual transition between the intact and the blind part of the visual field was confirmed according to the clinician's evaluation. Patients were excluded if they had at least one of the following symptoms: malignant brain tumor, eye or other central nervous system diseases, electric or metallic implants in the eyes or head, expected low compliance, history of epileptic seizures within the last 10 years, or use of antiepileptic or sedative drugs during the recruiting process. On admission to the study, the medical history was collected and assessed by a neurologist. A comprehensive examination, in particular of visual dysfunction, was carried out. The possibility of further participation in the study depended on the results of this preliminary investigation. The patients had to have some residual visual performance, evident by a gradual transition between the blind area and the visual field's intact area.

### Experimental Design

#### tDCS/tACS

The tACS and cathodal tDCS stimulation was delivered with conductive-rubber electrodes placed in saline-soaked sponges and connected with a NeuroConn MC8 stimulator. The tACS stimulation electrode (5 × 7 cm) and a reference electrode (10 × 10 cm) were placed at Fpz and at the right upper arm, respectively, according to 10–20 system EEG recordings. Stimulation started with a 5-Hz burst, and then frequency increased in steps of 1–30 Hz using a 48-s-long “rtACS block.” The tACS stimulation was given for 20 min per day with a maximum current of 1.5 mA (peak-to-peak), which was well above the phosphene threshold (47). The block was repeated for 20 min. In the tDCS condition, the cathode was positioned above the intact hemisphere, and stimulation was done for 10 min immediately before rtACS and set at 1 mA using one electrode placed at either O1 or O2 position (3 × 3 cm) with anode at Fpz. The impedance was kept below 10 kΩ.

#### Sham Design

Sham patients had the identical electrode montage and stimulation duration. The tACS sham condition was designed to induce (short-lasting) phosphenes that patients could subjectively report (47); that is, it was a minimal stimulation. In addition, occasional current bursts were given to create short but presumably therapeutically ineffective phosphenes (45) involving one 5-Hz burst/min with individual amplitude for phosphene perception as used in a previous study where none of the subjects could tell to which group they belonged (45). In contrast, the tDCS sham-condition was designed to elicit only cutaneous sensations that gradually disappear because of habituation (48). Here, the current was ramped up for 30 s, then stopped, and at the end of the session ramped down for another 30 s as shown in Figure 1A (45). Through this design, we ensured that all patients felt their skin comparable in degree and duration with the active tDCS. The combined tDCS/tACS stimulation was designed to indicate whether prior cathodal tDCS on the intact hemisphere (a kind of conditioning) could enhance rtACS effects compared to sham stimulation and rtACS without preceding tDCS (45). Here, cathodal tDCS was applied to reduce the interhemispheric imbalance by inhibiting the visual cortex of the intact hemisphere (Figure 1B). All sham patients had been offered to receive stimulation treatment after the final follow-up evaluation. The stimulation parameters were kept unchanged for 20–30 min per day during the 2 weeks' treatment (Figure 1C). Of note: for all stimulation conditions, the default setting of the neuroConn stimulator gives short pulse of 50 Hz at 0.5 μAmp every 2 s to monitor the skin resistance.

#### Safety of Electrical Current Stimulation

The relatively large surface area of electrodes during stimulation limited the maximum threshold of current densities compared with other studies. The maximum current density was 42 μA/cm^2^ below AC stimulating electrodes and 15 μA/cm^2^ below the reference electrode. In the case of tDCS, it was 111 μA/cm^2^ below the stimulating electrodes, which corresponded to a total charge density lower than 0.1 C/cm^2^, which was below the safety limits as described in the previous study (22). Safety guidelines for direct current applied to the human brain were reported (16, 22, 49).

The following undesirable events had been observed immediately after each stimulation session and the following day before the next stimulation session: rare cases of headache, dizziness, fatigue/drowsiness, skin sensation, blurred vision immediately after stimulation, and others. Patients were not asked to perform a visual task during stimulation sessions but just kept their eyes closed while sitting down.

### EEG Recording and Pre-processing

High dense array EEG was recorded using a HydroCell GSN 128-channel net and Net Amps 300 amplifier (EGI Inc., Eugene, OR, USA) with sampled frequency 500 Hz. Impedance was ascertained to be <50 kΩ throughout the recording. Patient's resting-state EEG was recorded at three time points (before treatment: Pre, after 10 days of treatment: Post, follow-up after 2 months: FU). During the recording, patients were instructed to keep relaxed, with their eyes closed, for at least 5 min. There was no significant difference in patient's age in the three group after a Kruskal–Wallis test (*p* > 0.05), ACDC (mean ± SD = 53.45 ± 14.18), AC (mean ± SD = 58.25 ± 9.54), and sham (mean ± SD = 63.87 ± 5.38).

EEG signals were analyzed with MATLAB version 2019a and Fieldtrip (50). A digital 1–145-Hz bandpass filter was applied as well as a 50-Hz notch filter, and the data were down-sampled to 250 Hz and then referenced by the common average reference method. Five-min-long EEG recordings for both groups were segmented into 2-s-long epochs with 0.5-s overlapping. Components of eye blinks and cardiac activity were removed by an independent component analysis algorithm. The frequency was decomposed in seven frequency bands: Delta (1–3 Hz), Theta (4–7 Hz), Alpha1 (8–10 Hz), Alpha2 (11–13 Hz), Beta1 (14–21 Hz), Beta2 (22–30 Hz), and the whole alpha band as (8–13 Hz).

### Source Construction

The forward model was calculated using the symmetric boundary element method (51); inverse model was calculated with a beam-forming method using the partial canonical correlation method (52), which implements dynamical imaging of coherent sources (53) algorithm for computing the spatial filters for each dipole location in the source model. The estimation of noise was projected with option cfg.projectnosie = “yes” in Fieldtrip toolbox to remove the center of the head bias with a regularization parameter λ = 5%. The Automated anatomical labelling-Volume-of-Interests atlas is an automatic anatomical labeling result (54) of spatially normalized, single-subject, high-resolution T1 MRI data set provided by the Montreal Neurological Institute (MNI) (55), which includes 120 structure definitions, only 90 subareas were used in this study.

### Functional Connectivity Estimation

Brain functional connectivity was assessed by calculating the statistical synchronization to quantify the interaction between different brain region pairs (56, 57). Functional connectivity was estimated with imagery part of coherence at the anatomical level. Coherence can be used to quantify how the brain regions synchronize neural oscillation among each other (58). This method is insensitive to false connectivity arising from volume conduction to measure the functional connectivity with resting-state EEG data (59). Both the sensor level and anatomical level were defined as follows:


$$\begin{array}{c} icoh_{\left(f,t\right)}=|im\left(\frac{\sum_{n\;=\;1}^{N}S_{1}^{n}\left(f,t\right)S_{2}^{n^{*}}\left(f,t\right)}{\sqrt{\sum_{n\;=\;1}^{N}|S_{1}^{n}{\left(f,t\right)}^{2}|\sum_{n\;=\;1}^{N}|S_{2}^{n}{\left(f,t\right)}^{2}|}}\right)| \end{array}$$

where $S_{1}^{n}\left(f,t\right)\;$ and ${\;S}_{2}^{n^{*}}$ (f,t) are the frequency-decomposed EEG data from two specific regions for every subject and condition. Coherence between all pairs of dipoles were parcellated with the AAL atlas with the imaginary part of coherence for each subject per frequency band to obtain a parceled connectivity matrix (90 × 90). Coherence was segmented into short (local) and long (global) range, and the local coherence was determined within each lobe of interest; the long-range coherence was from left or right occipital (LO or RO) compared to the rest of the brain regions [RO] to [RT, RF, RP, LT, LF, LP, LO] or [LO] to [RT, RF, RP, RO, LT, LF, LP].

In our analysis, we used graph theory, which was developed to analyze complex network structures. It is a method now widely used to explore brain functional connectivity (FCN) changes (60, 61). Graph theory describes important properties of brain networks by quantifying typologies of their respective network measures by anatomical tracts or by functional associations (62). According to graph theory, brain areas are referred to as “nodes” or “vertices,” and edges represent the connections between the nodes. The term “node degree” is used for the number of links connected to a center node, and strength is the sum weights of links connected to the center node. The clustering coefficient is the fraction of triangles around the center node (61). Node betweenness centrality is the value of all shortest paths that pass through a given node. Nodes with high values of centrality involve a large number of shortest paths. The key features of a network structure are the clustering coefficient and the path length of its connections. While a high cluster coefficient is a sign of a rather stable network, a short path length is less stable but more flexible. Brain network structures are typically somewhere in between these two poles. It is a compromise of both poles, stability and flexibility, and is called a “small world” network. The network structure was defined by different as now described.

#### Characteristic Path Length

In the graph theory, the shortest path length is the short distance from one node to another node, which related to network efficiency and information transfer rate (61); the characteristic path length (CPL) is the average shortest path length of all nodes in the network with a definition:


$$\begin{array}{c} L\;=\;\frac{1}{n}\underset{i\in N}{\sum }L_{i} \end{array}$$

where *l*_*i*_ is the average path length of between node *i* and all other nodes.

#### Clustering Coefficient

The clustering coefficient is the fraction of triangles around a node and is equivalent to the fraction of the node's neighbors that are neighbors of each other (63).


$$\begin{array}{c} C\;=\;\frac{1}{n}\underset{iN}{\sum }C_{i}\;=\;\frac{1}{n}\underset{iN}{\sum }\frac{2t_{i}}{K_{i}\left(K_{i}-1\right)} \end{array}$$

where *C*_*i*_ is the clustering coefficient of node i (*C*_*i*_ = 0 for *K*_*i*_ <2).

### Visual Field Diagnostic

Visual field parameters (visual field: FOV, high-resolution perimetry [HRP]) were assessed in patients to quantify the visual impairment in different phase. The contralateral eye's FOV was measured by OCULUS Twinfield®. HRP demonstrates the visual field charts generated by high-resolution computer-based perimetry developed by the Sabel laboratory (1).

### Data Analysis and Software

Data analysis was conducted with MATLAB, 2017a (64). EEG was preprocessed and resourced in Fieldtrip (50), the functional connectivity measures were calculated by the brain connectivity toolbox (61), and the long coherence was visualized by BrainNetViewer (65). Pearson correlation was performed between the behavior data and brain network measures at each frequency band. Because our study was explorative, no adjustment was made for multiple comparisons (66).

Visual fields were analyzed with respect to absolute change in HRP and percentage change in FOV after NIBS per group. A repeated-measures analysis of variance (ANOVA) test was performed (three groups: ACDC, AC, and Sham, and two time periods: post–pre and FU–pre). *p*-value was corrected by the Tukey–Kramer test in the *post-hoc* analysis.

## Results

### Visual Field Recovery

A detailed description of visual field recovery is published elsewhere (35). However, to explore the functional meaning of brain network changes, here we report detection performance in the visual fields and the RTs.

*Visual field:* There was no significant main effect (*F*_(1, 21)_ = 0.002, *p* = 0.9) and no interaction effect (*F*_(1, 21)_ = 0.46, *p* = 0.63) in visual field detection performance. However, as shown in Figure 1D, the ACDC group's FOV increased after treatment, and this was maintained at follow-up. In contrast, the other two groups' median FOV remained unchanged after treatment and at follow-up. This suggests that visual functionality of the ACDC group had a trend of an enhancement at a group level compared with baseline, which was not observed in the other two groups.

*Reaction time*: No significant interaction effect was observed [*F*_(1, 21)_ = 1.49, *p* = 0.24] on white and gray RT percentage shown in Figure 1D. However, there was a trend of ACDC RT decrease (which is an improvement) in both Post and FU in the intact sector of the visual field. In contrast, both AC and Sham groups' RT increased in Post and FU compared with baseline. As for the gray area, there was neither significant interaction [*F*_(1, 21)_ = 0.006, *p* = 0.99] nor a main effect for the group interactions [*F*_(1, 21)_ = 0.84, *p* = 0.37]. However, RT of ACDC decreased, while the RT of AC and Sham increased comparing with baseline in Post and FU. This indicates the ACDC group has a greater visual acuity percentage change than the other two groups.

#### Brain Network After Brain Stimulation

**Figure 2:**
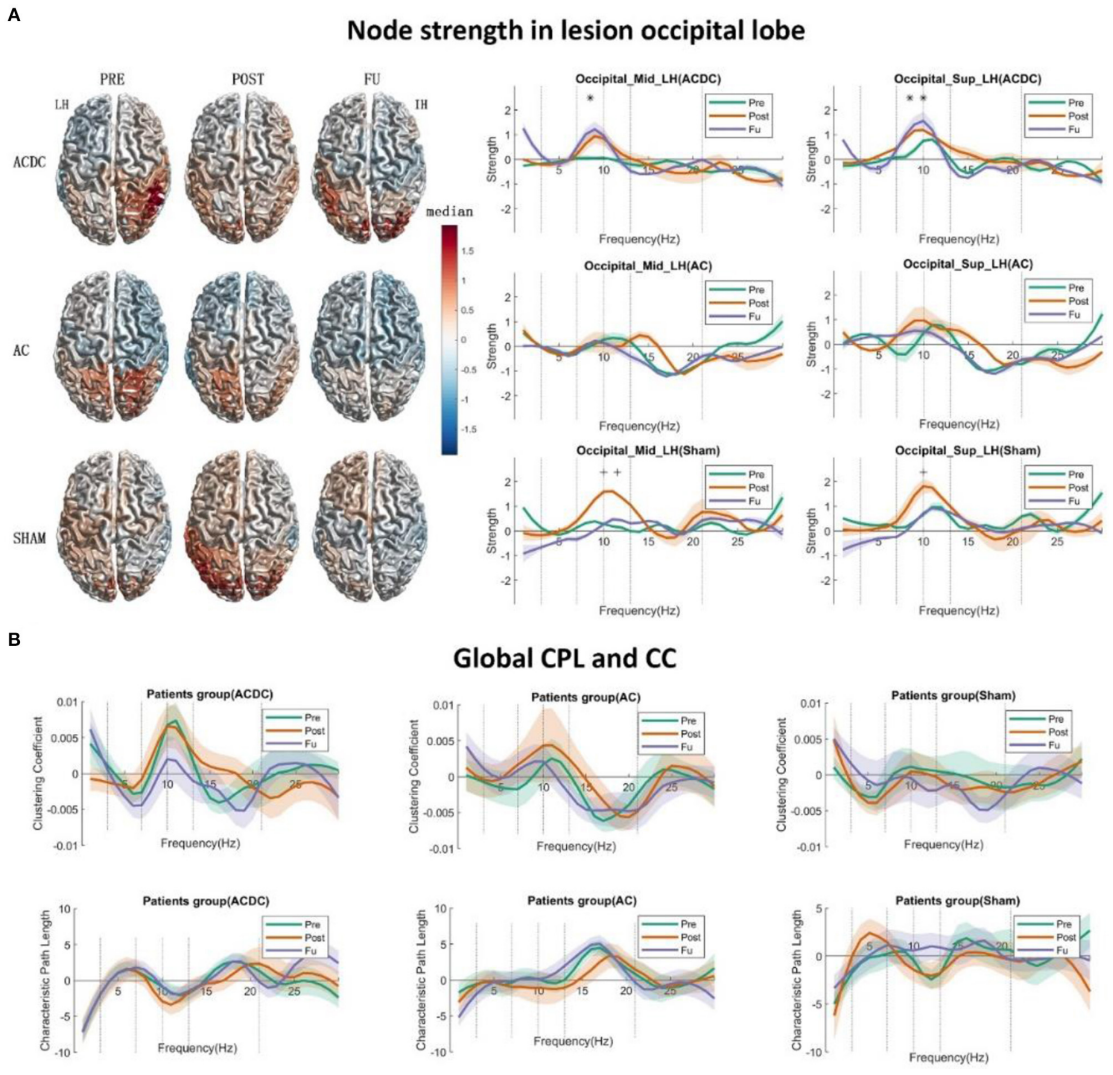
**(A)** The left side displays the surface plot of the median node strength per group in the alpha band. Baseline (PRE): ACDC and AC groups have stronger connectivity than the SHAM group in the intact hemisphere (parietal and occipital), after treatment (POST): ACDC and AC groups have lower node strength than the SHAM group (parietal and occipital). Follow-up (FU): ACDC has stronger connectivity than the AC and SHAM group. Right part: Line plot of the single occipital lobe from 1 to 30 Hz with control baseline corrected. In ACDC, the middle and superior occipital regions have greater strength at POST and FU than PRE (*p* < 0.05). Meanwhile, the AC group shows a similar pattern for a three time points (*p* > 0.05) in the above two ROIs. The SHAM group's node strength was significantly enhanced after treatment and then dropped down to the original level. **(B)** This shows the global clustering coefficient and CPL for three groups. The ACDC group had decreased CC at follow-up, indicating that information transfer in the whole brain needed to pass fewer nodes than baseline (i.e., it is more efficient). Here, the connections are more ordered or less diffuse. No significance was observed in the other groups. *p < 0.05.

We performed a two-way 3 (stimulation group: ACDC, AC, and sham) × 3 (time: Pre, Post, and FU) mixed-design ANOVA with repeated measures on the time variable of local node strength and long coherence (Figure 2A). A compound symmetry assumption was checked before statistical analysis was performed. The regular *p*-value calculations in the repeated measures were reported if the theoretical distribution of the response variables was of the same variance. *p*-value calculations were corrected with Greenhouse-Geisser approximation. The *post-hoc* test was estimated with a significant sign (*p* < 0.05) after a mixed-design ANOVA test, and the family-wise error rate was controlled by the Tukey–Kramer test after estimating homogeneity of variances.

To explore the role of brain functional network reorganization as potential mechanisms of recovery, we calculated the two global parameters CPL and “global clustering coefficient” using a 30% threshold of the connectivity matrix (67).

#### Between-Group Analysis

There was no significant interaction effect on the alpha band in the occipital lobe.

#### Within-Group Analysis

A significant main effect of strength was observed in the ACDC group with repeated time measures on occipital_sup of the lesioned hemisphere [*F*_(2, 42)_ = 5.31, *p* = 0.009]. We ascertained that the assumption of sphericity was not violated (*W* = 0.97, *p* = 0.74). Thus, in the ACDC group, the strength of three treatment time points on occipital_ sup_LH differed significantly. *Post-hoc* analysis showed that FU strength on occipital_sup _LH was significantly higher than Pre (median ± SEM = 0.84 ± 0.32, *p* = 0.044).

The significant main effect of strength in Sham group was observed with repeated time measures on occipital_mid of the lesioned hemisphere [*F*_(2, 42)_ = 4.486, *p* = 0.017] and occipital_sup of lesioned hemisphere [*F*_(2, 42)_ = 5.31, *p* = 0.009]. The assumption of sphericity was not violated (*W* = 0.99, *p* = 0.98; and *W* = 0.97, *p* = 0.74, respectively). This shows that if we only consider the sham group's treatment, the strength of three time points on occipital_mid_LH and occipital_sup_LH significantly differed. *Post-hoc* analysis showed that the node strength of occipital_mid_LH after Sham treatment was significantly higher than before treatment (median ± SEM = 1.01 ± 0.42, *p* = 0.050) and follow-up (median ± SEM = 1.35 ± 0.39 *p* = 0.007). Moreover, the occipital_sup_LH node strength after Sham treatment was also observed to be significantly lower than follow-up (median ± SEM = 1.30 ± 0.41, *p* = 0.011) (Figure 2A, right part).

#### Global Small World Networks

According to graph theory, a network structure can be characterized by two opposing poles: a high cluster coefficient with long path length (an “ordered” network) and a low clustering with short path length (a “random” network). If the network is in between those two poles, it has a proper balance between “stability” and “efficiency.” Then it is called a small world network. Patterns of anatomical connectivity in neuronal networks are sometimes characterized by high clustering and a small path length (63). We calculate the global CPL and CC using the 30% threshold of connectivity matrix as a criterion for three treatment groups to identify the small world network dynamic changes. Using these network parameters, we performed a two-way 3 (group: ACDC, AC, and sham) × 3 (time: Pre, Post, and FU) mixed-design ANOVA with repeated measurements on the time variable. The compound symmetry assumption was checked before statistics was performed. The regular *p*-value calculations in the repeated measures were reported if the theoretical distribution of the response variables had the same variance, provided the compound symmetry assumption was not violated; *p*-value calculations were corrected with the Greenhouse–Geisser approximation, and the *post-hoc* test was estimated with a significance level of *p* < 0.05 after a mixed-design ANOVA test. The family-wise error rate was controlled by the Tukey–Kramer test following estimation of the homogeneity of variances. No significance was observed for the global CPL and CC (Figure 2B). However, a trend was noted in that the global CC of ACDC was decreased in FU, whereas the global CPL remained at the same level as before, a clear sign of a more efficient small-worldness network after ACDC treatment.

#### Correlation Between Brain Network Measure and RT

**Figure 3:**
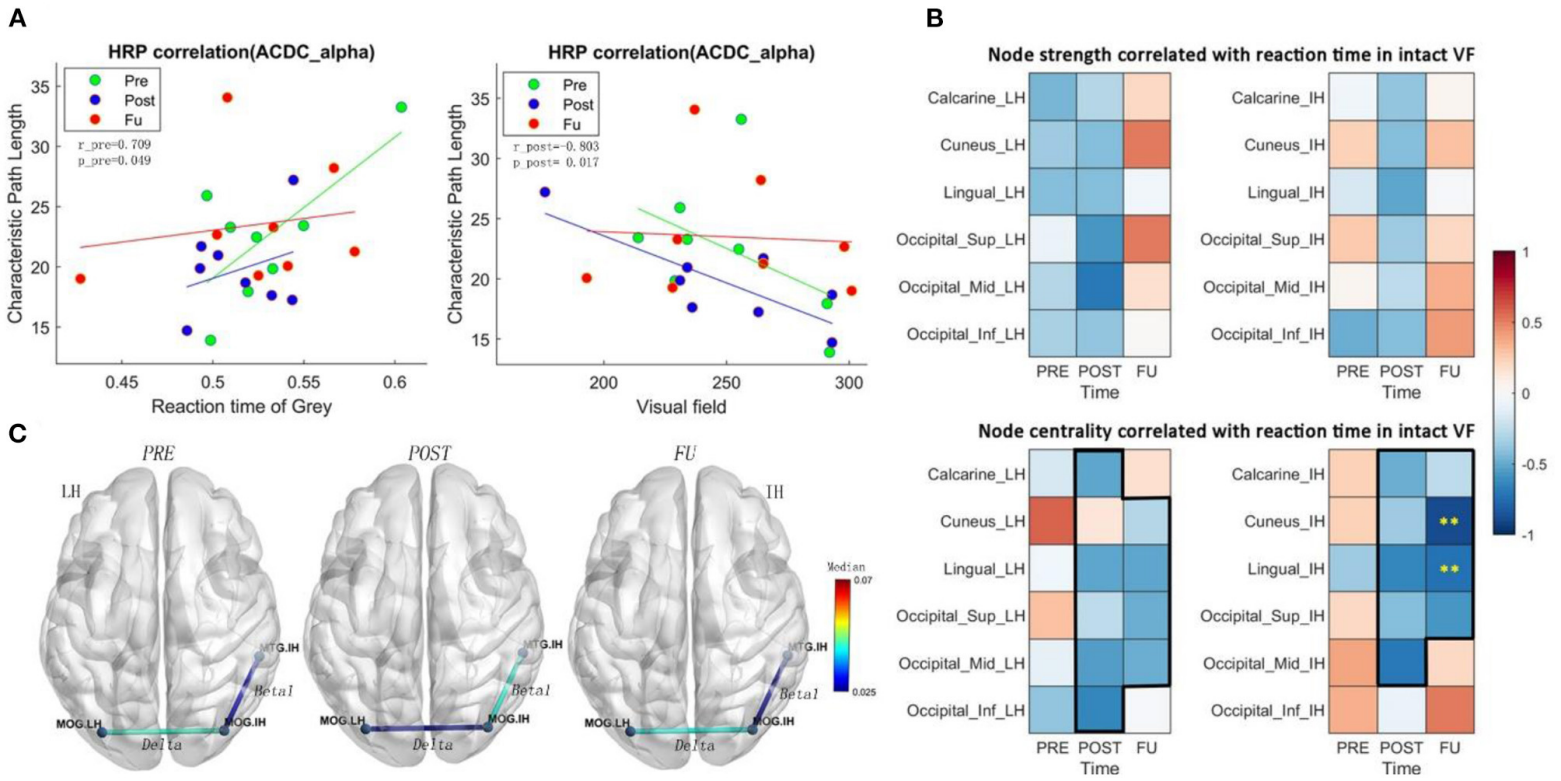
**(A)** CPL in the alpha band of ACDC patients correlates positively with RT of residual visual area before treatment and correlates negatively with the intact visual field after treatment. It indicates that the larger the intact visual field in the ACDC group after treatment, the lower is the CPL in the ACDC group in the alpha band. A lower CPL is a sign of decreased average shortest path length in the resting state of brain functionality. When neural processing in the brain has a lower CPL; this can be interpreted as a biomarker that brain process is globally enhanced and more efficient. **(B)** Heat map showing correlation coefficients (ranging from −1 to +1) between brain node measures and reaction time in the intact VF of the ACDC group. The upper panel shows a trend of a treatment effect in the intact hemisphere (IH) and the lesioned hemisphere (LH) in both hemispheres; the lower panel shows a clear pattern after treatment: at follow-up, faster reaction time was associated with higher brain node centrality. Significant negative correlation was observed in cuneus and lingual in the IH. This could indicate that the visual function recovery after brain stimulation could be due to the multifactor integration of the lesioned and the intact hemisphere. **(C)** ACDC significantly reduced the long coherence between the lesion and intact occipital cortex in the delta band after POST (*p* < 0.05). Also, low beta was enhanced after POST between the intact occipital and intact temporal (*p* < 0.05). Each connectivity was measured between LH and IH occipital lobe; LH, lesion hemisphere; IH, intact hemisphere; MTG, Temporal_Mid; MOG, Occipital_Mid; LO, lesion Occipital_Mid lobe; IO, intact Occipital_Mid lobe; IT, intact Temporal_Mid lobe.

The functional meaning of the network dynamics can be explored with correlation analyses. We found a negative correlation between the intact visual field and CPL (global CPL), which was significantly different at post (*r* = −0.80, *p* = 0.017) in the ACDC group (Figure 3A); this indicates that a larger visual field is associated with lower CPL after treatment. Furthermore, a positive correlation was observed between RT in areas of residual vision and CPL at Pre (*r* = 0.70, *p* = 0.049), suggesting that slower RT of the residual visual field was associated with higher CPL.

The correlation between brain network measures at subregions of occipital lobe and RT in intact VF shows a trend of a treatment effect as well. Node strength correlated negatively with both the intact and the lesioned hemisphere (Figure 3B), indicating that better visual function is supported by higher node strength in the brain. As for centrality, which is a measure to quantify how many shortest path length go through one node, the results show that after treatment and follow-up better visual function is supported by a state where brain has a higher capacity to compensate spontaneously.

The centrality of cuneus (*r* = −0.88, *p* < 0.01) and lingual (*r* = −0.73, *p* < 0.01) in the intact hemisphere was significantly correlated with the RT (Figure 3B), demonstrating that ACDC may have long-lasting modulation effect than the other two groups (see follow-up). ACDC enhances both hemispheres' brain connectivity, and especially, we could assume that “silent” neurons were reactivated, more functional connectivity could be rescheduled and transferred around the lesion part.

#### Global Brain Connectivity

The connection coherence from lesion occipital and intact occipital to other brain regions was calculated, as shown in Figure 3C. A two-way mixed-design ANOVA test was conducted on brain network measures between group (ACDC, AC, sham) and time (before treatment: Pre, after treatment: Post, and: FU). The *post-hoc* analysis has been performed for the pairwise comparison with a significant level of *p* < 0.05; the family-wise error rate was adjusted.

A significant coherence was observed between the lesioned occipital (LH) and the intact occipital (IH) region in three measurements (*F*_(2, 42)_ = 6.509, *p* = 0.003), and the neural correlation in the delta band was significantly declined between the lesion occipital and intact occipital lobe after ACDC. *Post-hoc* analysis indicates that coherence after Post was lower than the Pre (MD ± SEM = −0.014 ± 0.005, *p* = 0.036), with a trend at FU (MD ± SEM = −0.016 ± 0.007, *p* = 0.069).

A significant coherence was also observed between intact occipital (IO) and intact temporal (IT) in three measurements (*F*_(2, 42)_ = 6.16, *p* = 0.004). The coherence between IO and IT was enhanced after Post and significantly declined at follow-up in the ACDC group in the low beta band. *Post-hoc* analysis indicates that the coherence of FU was lower than at Post (MD ± SEM = −0.017 ± 0.006, *p* = 0.018). And there was a trend of coherence enhancement after Post when compared with Pre-treatment (MD ± SEM = −0.018 ± 0.007, *p* = 0.054).

### Comparing Responders With Non-responders

To further clarify the role of brain FCN reorganization in vision recovery and validate the meaning of our correlation results, we compared responders and non-responders. To this end, we used the contralateral visual field as obtained by standard Oculus perimetry as a criterion to classify each patient as either a responder or non-responder, irrespective of which treatment they received. Therefore, here we calculated only the correlation between FOV and EEG measures to compare responders and non-responders.

**Figure 4:**
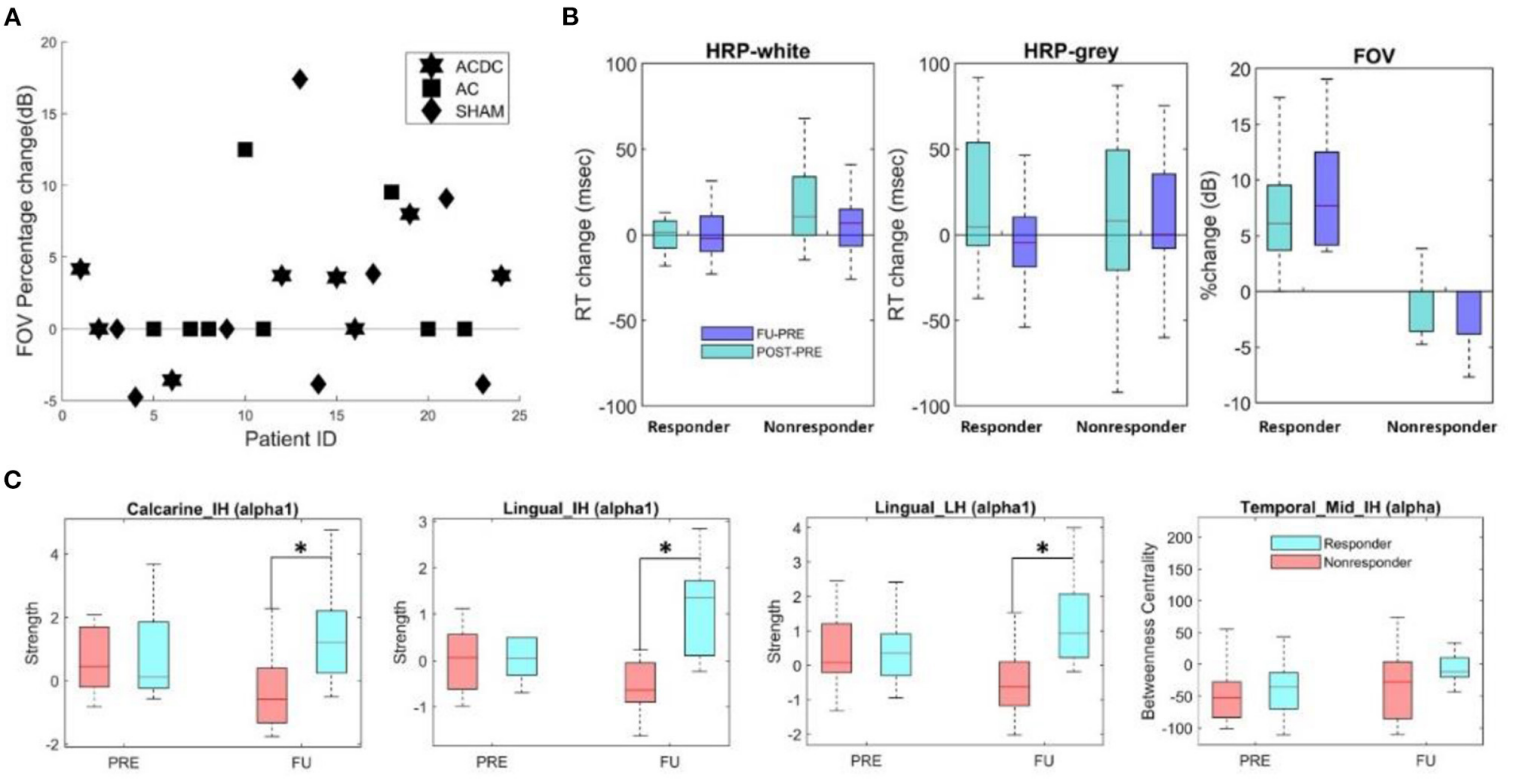
**(A)** Percentage change of FOV per group for responders with values above zero (*n* = 10) and non-responders (*n* = 14). **(B)** Boxplot of absolute median change (HRP) and percentage of FOV (unit: dB) in responder and non-responders. Both groups had comparable reaction times (RT), but (per definition) the percentage change of FOV was significantly higher (*z* = 4.17, *p* < 0.001). **(C)** The local node strength in the low alpha band was significantly enhanced in responders in calcarine and lingual areas of both hemispheres (*p < 0.05). The FU centrality of Temporal_Mid_IH was higher than the PRE (*p* > 0.05).

As shown in Figure 4A, patients above the zero line were considered responders (*n* = 10) and all other non-responders (*n* = 14). We performed a two-way ANOVA test (group: responder and non-responder, time: Post vs. Pre, FU vs. Pre) to investigate HRP changes that were not, but the Mann–Whitney *U*-test revealed that the FOV was greater in the responders (*z* = 4.17, *p* < 0.001) at Figure 4B (right part). Of note, this difference was the result of the definition of responder and confirms that both groups are, in fact, different. It does not demonstrate treatment efficacy.

#### Local Brain Network Dynamics Changes

To compare the local node strength and centrality changes in responders with non-responders, we performed a two-way repeated ANOVA (groups: responder and non-responder, time: baseline and FU). In the lesioned hemisphere, the low alpha-band node strength in calcarine (*F*_(1, 22)_ = 6.42, *p* = 0.018) and lingual lobes (*F*_(1, 22)_ = 7.38, *p* = 0.012) was significantly different between responders and non-responders during follow-up. The *post-hoc* test showed in responders higher node strength in calcarine (MD ± SEM = −1.56 ± 0.57, *p* = 0.013) and lingual area (MD ± SEM = −1.68 ± 0.49, *p* = 0.002) (Figure 4C). In the intact hemisphere, both low alpha band node strength in calcarine (*F*_(1, 22)_ = 9.60, *p* = 0.005) and lingual lobes (*F*_(1, 22)_ = 5.76, *p* = 0.025) was significantly different between both groups during follow-up. *Post-hoc*, they were higher in responders for node strength in calcarine (MD ± SEM = −1.56 ± 0.65, *p* = 0.026) and lingual (MD ± SEM = −1.503 ± 0.427, *p* = 0.002). No significance was observed for brain network measure centrality. However, centrality of intact middle occipital at FU was lower than Pre in responders (*p* = 0.32, MD = −44).

#### Global Network Measures

**Figure 5:**
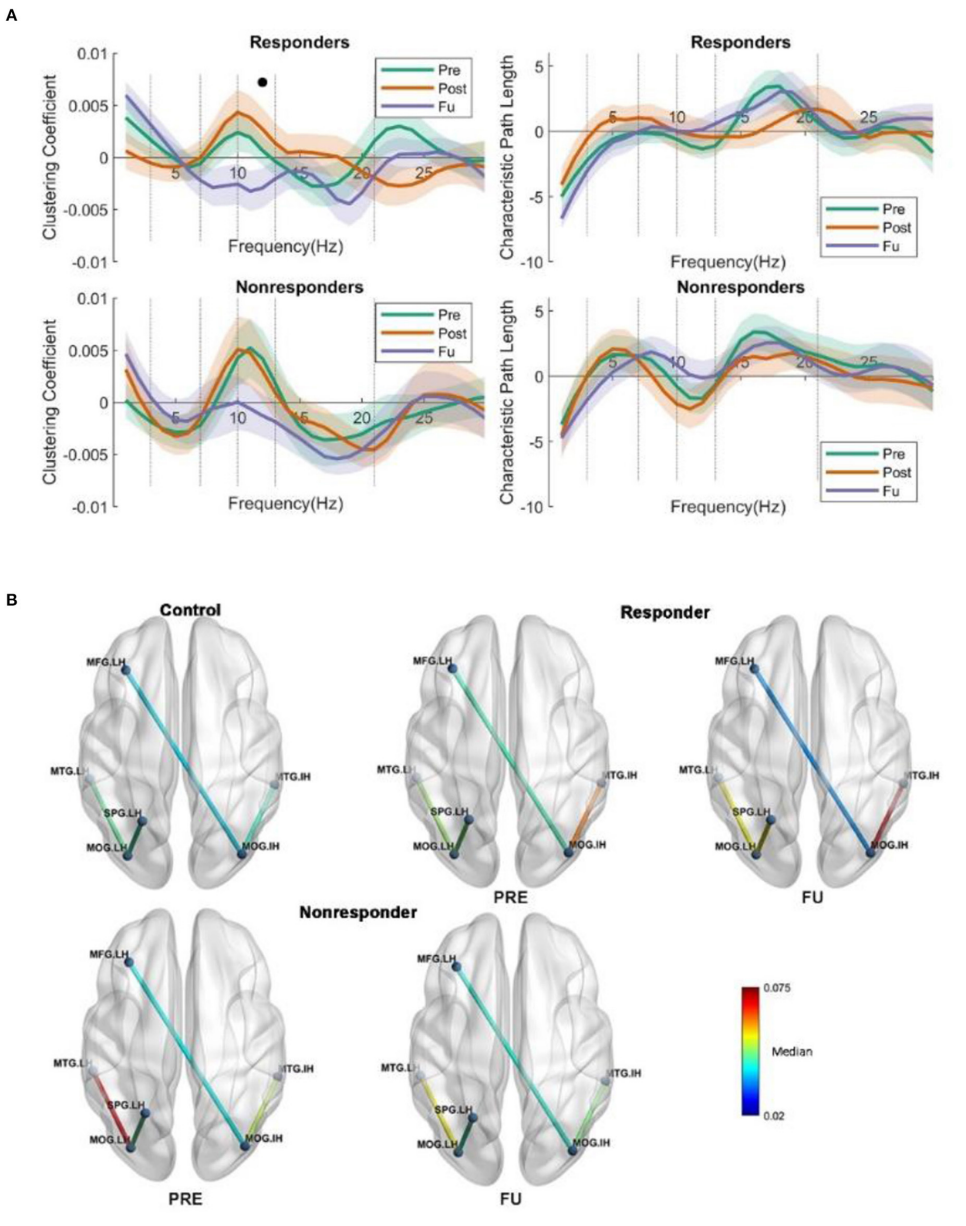
**(A)** Global clustering coefficient and CPL show no interaction or main effect for characteristics path length (two groups/three time points). But a main effect was observed in the responders when comparing FU and POST, namely, a lower clustering coefficient. **(B)** The global coherence. From **(B)**, we could see the strength of the intact middle occipital increased, whereas the centrality decreased, which is very interesting; we may suggest that intact middle occipital gets rid of redundant connections from various regions but enhanced the connection with intact temporal lobe, as the temporal lobe could help the vision loss patient to handle the daily perception or movement identification. The long coherence between lesion frontal and intact occipital was observed significantly reduced at FU; the coherence was lower than that in PRE in the alpha band. The coherence between the lesion temporal and lesion occipital was significantly changed; both the responders and non-responders show higher coherence than the control subject.

Global network features are those that describe the state of the whole brain, irrespective of region. We first calculated the global clustering coefficient and global characteristic path length, followed by two-way repeated ANOVA (two groups/three time points). The *p*-value was corrected by the Tukey test in *post-hoc* analysis. The only significant finding was that global CC in FU was significantly lower than the Post (MD ± SEM = −0.0068 ± 0.0027, *p* = 0.05) in the high alpha band (Figure 5A). This suggests that responders need fewer connections to handle the neuronal synchronization in the resting state.

#### Global Coherence for Responder and Non-responder

To investigate the long coherence fluctuation irrespective of the stimulation protocols, a two-way repeated-measure ANOVA was performed (three groups: control, responder, and non-responder, and two time points: Pre and FU). The *p*-value was adjusted for multiple comparisons by Tukey–Kramer test for *post-hoc* analysis.

A main effect on coherence was observed between intact occipital and lesion frontal in the alpha band (*F*_(1, 45)_ = 4.032, *p* = 0.05) (Figure 5B). *Post-hoc*, the FU coherence between the occipital_IH and frontal_LH was significantly lower than baseline in responders (median ± SEM = 0.0069 ± 0.003, *p* = 0.025), and an interaction effect was observed when investigating the difference between the control and responders during FU (*F*_(2, 45)_ = 4.04, *p* = 0.024) in the alpha band. Furthermore, at FU, the coherence between the occipital_IH and temporal_IH was significantly higher in non-responders when compared to controls (MD ± SEM = 0.0140 ± 0.0053, *p* = 0.030).

#### Correlation Between FOV and Brain Network Measures

**Figure 6:**
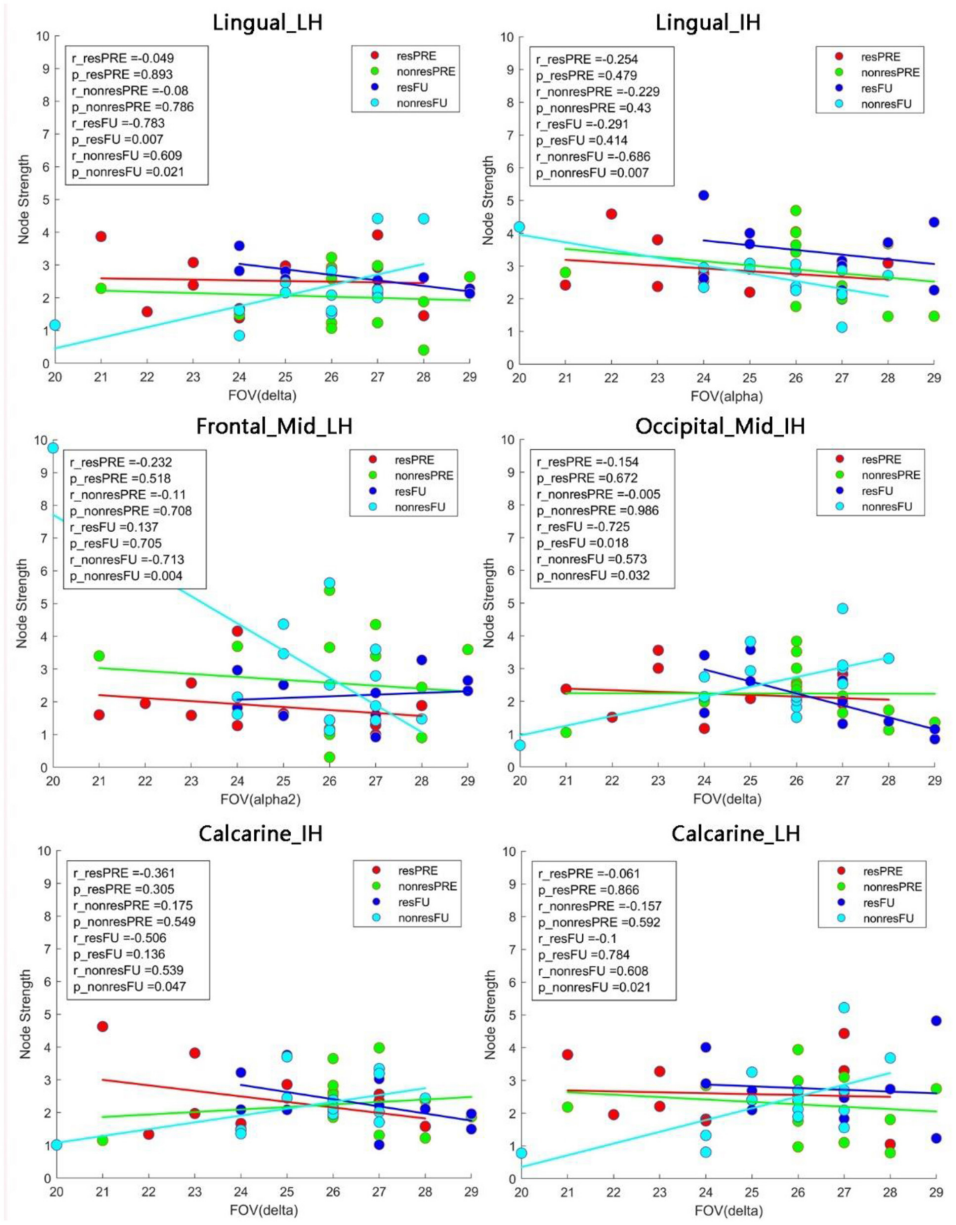
Pearson correlation between the FOV and node strength. In non-responders, we observed a positive correlation between FOV and node strength in the lingual_LH and calcarine_LH in the delta band. In the alpha band, node strength of both the lingual_IH and middle frontal_LH was negatively correlated with FOV. resFU, responders at FU; nonresFU, non-responders at FU; resPRE, responders at baseline; nonresPRE, non-responders at baseline.

**Figure 7:**
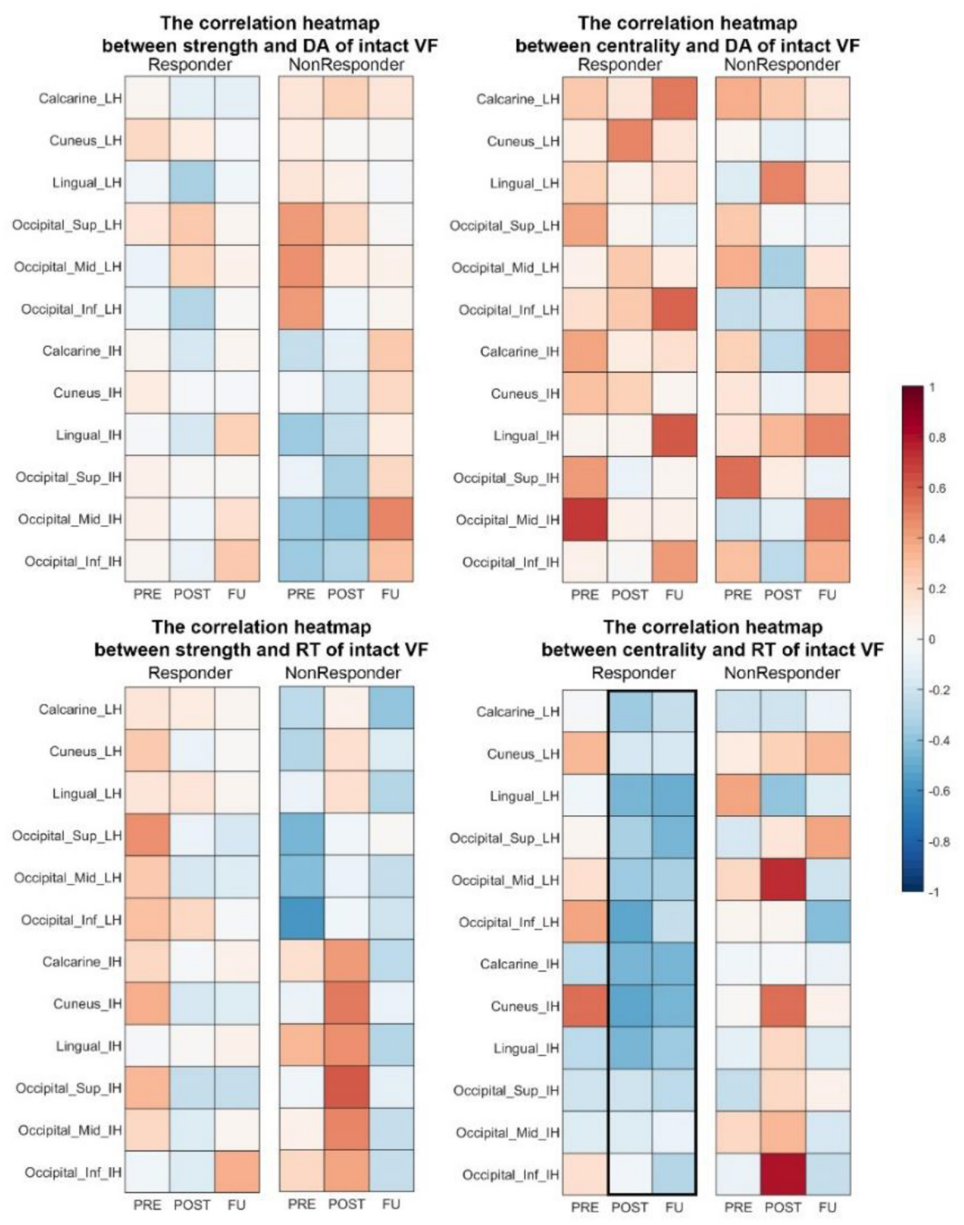
Correlation heat map between brain network measures (strength and centrality) and visual performance in responders. At POST and FU, there was a trend of faster RT being associated with higher centrality in both hemispheres. DA, detection accuracy; VF, visual field; RT, reaction time.

Pearson correlations were calculated to investigate the relationship between visual functionality measurements (FOV: Figure 6 and HRP: Figure 7) and node strength. We observed significant correlations both in the lingual_LH (r_resFU = −0.783, *p* = 0.007) and middle occipital_IH (r_resFU = −0.725, *p* = 0.018), where responders with higher FOV showed lower node strength in the delta band and higher node strength in non-responders in lingual_LH (r_resFU = 0.609, *p* = 0.021) and middle occipital_IH (r_resFU = 0.573, *p* = 0.032). This suggests that in both hemispheres, responders with higher FOV had lower delta band strength, whereas non-responders had higher delta band strength.

The same delta band pattern was also noted in both hemisphere of calcarine (lesion hemisphere: r_nonresFU = 0.608, p_nonresFU = 0.023, intact hemisphere: r_nonresFU = 0.539, p_nonresFU = 0.047) in non-responders. Delta band node strength was positively correlated with FOV in non-responders. This may be one possible reason why non-responders failed to improve their vision because of the delta band oscillation in visual cortex.

High alpha-band node strength correlated significantly with FOV in lingual_IH (r_nonresFU = −0.686, *p* = 0.004) and middle frontal_LH (r_nonresFU = −0.686, *p* = 0.007) in non-responders. This indicates that alpha-band node strength decreases with higher FOV measure in non-responders.

## Discussion

To study brain FCN reorganization following NIBS in hemianopic stroke, we used graph theory to analyze the local and global network features and how they correlate with visual field recovery. The study's aim was to find a stimulation protocol for clinical use in stroke rehabilitation.

### Behavioral Performance

We studied different visual field parameters (FOV and HRP), which were already reported in detail elsewhere (35). For the present study, FOV and HRP data were used to establish correlations with FCN parameters (Figures 1D, 4A,B; Table 1). As we showed, ACDC patients showed only a trend of an improvement over baseline of FOV and faster RTs. In contrast, AC and Sham patient at Post and FU showed a slower RT and no change over baseline of the FOV. But the combined tACS and tDCS enhanced visual performance compared to baseline, which was not observed in the other two groups. The output of observable behavior performance enhancement of visual functions was ACDC >AC > Sham. Because only the ACDC had improved visual performance, this raises the question as to possible brain FCN reorganization in this group.

### Local and Global Network Alteration After NIBS

Neuroplasticity is a critical factor in many neurological or neuropsychiatric diseases (29). Hence, modifying cortical activities by NIBS might be a promising therapeutic approach for clinical application (68). For example, tDCS shifts the suprathreshold of the resting state membrane potentials toward depolarization or hyperpolarization (69). Another approach is tACS, which entrains the neural oscillation in a frequency- and phase-specific manner (23) and induces an endogenous network coupling or decoupling in a long-lasting manner (70, 71). Vision loss in the blind is induced not only by primary tissue damage but can also be interpreted by a breakdown of synchronization in brain networks (72). Here, the intact hemisphere hyperactivity may be a possible mechanism of spontaneous compensation (73, 74), which may—or may not—be beneficial for neuron rehabilitation. In fact, compensation could be a possible barrier for recovery of the disturbed balance between both hemispheres, because hyperactivity of the intact hemisphere has the potential to inhibit the lesioned hemisphere's residual function (39, 40). We chose to test tDCS protocols because they allow for lateralized anode and cathode positioning tDCS protocols to modulate cortical imbalance between excitation and inhibition (45). But we also studied tACS, which allows manipulation of phase coherence between distant brain regions. The long-lasting effects of tACS were not only studied in optic nerve patients (see Introduction) but also reported in cognitive tasks (19, 75–78). Based on the above, the active tACS electrode was positioned at Fpz to entrain intrinsic neuron oscillation with a frequency of 5 to 30 Hz. These montages were tested in the present randomized and sham-controlled clinic trial using a sequential approach of tDCS followed by tACS.

#### Interhemispheric Balance After NIBS

Cortical network reorganization after an injury is a widely recognized phenomenon (79). One study reported hemianopia patients with damage on the left primary visual cortex who showed greater activation on other lobes in the lesion hemisphere and intact hemisphere associated with the visual cortex (80). Another study showed that bilateral SMA activation was increased after intensive rehabilitation of postural balance (81). Yet, others suggested that plastic reorganization of cognitive resources serves to compensate for impairment in stroke patients during motor rehabilitation tasks (82). However, such studies of cortical balance and recovery using EEG were not carried out with hemianopic stroke patients. Most recently, another study reported that vision restoration training can improve visual field defects in chronic hemianopia and that this correlated with functional brain network reorganization as measured by MRI in precuneus, which may help quantify patient's ability to direct spatial attention (83). In the present study, we first applied the cathodal tDCS over the intact visual hemisphere with the goal to inhibit the increased excitability caused by brain network reorganization after the stroke, which was then immediately followed by tACS applied at Fpz to entrain oscillations for both hemisphere. In the ACDC group, the middle and superior occipital lobe of the lesion hemisphere had significantly higher strength in Post and FU compared to baseline and showed greater network strength in the intact middle occipital lobe. An enhancement of the lesioned hemisphere in the ACDC group was also observed in the superior occipital lobe. The strength of both AC and sham group fell back to slightly below the original level during follow-up. Both occipital lobes' enhanced strength could demonstrate that interhemispheric connections were more balanced in the ACDC group. Because this correlated with visual performance in the ACDC group, this observation is compatible with the hypothesis that postlesion FCN plasticity reduces the imbalance between the lesioned and the intact hemisphere. As we showed, the unique protocol design of ACDC is able to modulate brain plasticity between the lesion and intact hemisphere, where the continuous stimulation can produce sustained and long-lasting neuronal modulation of the brain's neural networks. However, in stroke patients, ACS alone is largely ineffective, except for a benefit of foveal sensitivity (35).

The strength of calcarine_IH increased in the ACDC group, whereas the other two groups did not show similar patterns. The same change was also observed in the responder group. The centrality of both calcarine_IH and lingual_IH positively correlated with the RT in Pre and correlated negatively with RT both in Post and RT. This shows that the ACDC modulation enhanced the efficiency of information transfer on these two brain regions, which is associated with recovery of visual function (faster RT).

Regarding the issue of global brain network modulation, a lower CC was observed only in the ACDC group compared to baseline, where ACDC reduced alpha-band whole-brain connections. Our interpretation is that reduced connections are a sign of greater efficiency of visual processing, where less connectivity (greater processing efficiency) could comprise a possible mechanism of visual recovery.

#### Correlation of Visually Guided Behavior and Global Network FCN Measures

CPL is the average shortest path length in the network; a lower CPL indicates that fewer intermediary nodes are needed to transfer information between two unlinked nodes. In this case, the efficacy within a network is considered to be high. We found a significant positive correlation between the gray dots' RT and alpha-band CPL in the ACDC group at baseline, which disappeared at follow-up. This suggests that vision processing after ACDC modulation needs fewer nodes, i.e., fewer steps to process neural information. In the ACDC group, a significant negative correlation was observed between the number of white dots in HRP and CPL after treatment. This also suggests that ACDC decreased the whole-brain alpha-band CPL, and this was associated with an enlarged visual field. In contrast, CPL shows a very low negative correlation at FU. In summary, we suggest that ACDC can enhance processing efficiency in the alpha band, which also contributes to vision recovery.

#### Global Coherence After Brain Stimulation

The changes of the brain network in global coherence at both Post and FU show that NIBS can trigger brain plasticity by altering functional interaction between multiple brain regions. Specifically, ACDC reduced the functional connectivity between the lesion and the intact occipital lobe in the delta band and enhanced the connectivity between the intact occipital and the intact temporal lobe in the low beta band. In contrast, in the sham and ACS groups, no significant network changes were observed. Therefore, the combination of tACS and tDCS is apparently able to modulate neural plasticity by increasing the efficiency of communication between remote regions of the brain, possibly by improving interhemispheric balance.

#### The Challenge and Efficacy of Sham and AC Design

The design of a proper sham condition is one of the biggest challenges in NIBS studies because NIBS can elicit cutaneous sensations that gradually disappear due to habituation, and tACS induces phosphene perception (47). We used 5-Hz burst/min current bursts rather than “no stimulation” in the Sham tACS group. The current level was ramped up for the 30 s, then stopped, and at the end of the session ramped down for another 30 s in the sham tDCS group (45). In this way, we ensured a comparable effect and duration of cutaneous sensations for all the patients during stimulation with this unique design. In the sham group, however, the strength of temporal_mid_IH increased significantly after 10 days of stimulation and at follow-up returned back toward baseline levels. This suggests that the sham condition was not neutral but altered the strength, which might mean that the temporal lobe is sensitive to slow burst current in sham tACS, although no long-lasting effect was observed. Yet, node strength in the occipital cortex did not change after ACS.

### Comparison of Responders and Non-responders

Compared to non-responders, responders had less gray and more white visual field sectors. This is in agreement with the hypothesis that “areas of residual vision” (gray sectors) can be activated, improving regions of partial vision (84). Most patients with residual structures and functions spared by the damage have such “gray” regions where function is neither completely lost nor normal (85). The faster RT of white and gray regions of the visual field demonstrates that visual processing was enhanced in responders compared to baseline. Responders had significantly higher FOV than non-responders, both after treatment and at FU. This raises the question how the local and global brain network compares between responders and non-responders, irrespective of their NIBS treatment.

#### Local and Global Network in Responders

The total group of responders (i.e., irrespective to which group each patient belonged) showed significantly enhanced strength in the low alpha band in the lingual and calcarine lobe of both hemispheres, which non-responders did not. The lingual gyrus located between the middle of the temporal lobe and occipital lobe is relevant for complex visual processing such as object shape and contour information (86, 87). The calcarine sulcus is mainly involved in the primary visual cortex (V1) with a role in early-stage visual processing, creating a bottom-up saliency map from visual inputs to guide the shifts of attention (88, 89). The strength enhancement in both hemispheres could be a sign of compensation following the occipital damage. Similarly, the strength of the middle occipital region of the intact hemisphere was enhanced. We conclude that the reorganization occurs in two hemispheres symmetrically as a consequence of the occipital lobe. The correlation between network strength and behavior performance indicates that the delta band and alpha band play a vital role in vision recovery. Possibly regions with less alpha and higher delta are less responsive to the NIBS-induced oscillations, at least with regard to behavior output.

While in the delta band of the lingual and calcarine node non-responders had higher connection weights with higher FOV values, responders had fewer connection weights with higher FOV. Thus, delta band connectivity might play a critical role in enhancing visual functions and be a possible recovery biomarker of brain network reorganization. The same was noted in the intact middle occipital lobe; in responders, better vision was associated with lower delta band connectivity strength of the intact middle occipital, whereas the non-responders had higher node strength. The pattern is consistent with the neural correlation between the intact and lesion occipital lobe in the ACDC group: here, a lower coherence in the delta band between two occipital lobes was associated with visual field parameter improvement. Similarly, the global CC of the responder group in FU was significantly decreased in high alpha band compared to baseline. Thus, this finding also suggests that responders (with better vision recovery) needed fewer whole-brain connections in the high alpha band.

#### Correlation Between the FOV and Network Measures

In non-responders, greater visual acuity correlated with lower strength and FOV in the alpha band, especially at the frontal and lingual region. We also observed that the correlation between intact occipital and lesioned frontal lobe was decreased in responders. Thus, a local and global pattern of decreased connections between the intact occipital cortex and the lesioned frontal cortex signal seems to be a physiological correlate of vision recovery, and it shows that the middle frontal lobe plays an important role as a visual information-processing bridge, which was weakened after ACDC treatment.

#### Coherence Between the Intact Occipital and Intact Temporal Lobe

In responders, the FU coherence between intact occipital and intact temporal lobe was significantly higher than at baseline. It is known that the temporal lobe is responsible for handling perception and movement identification. Therefore, an enhanced connection between two lobes following NIBS suggests that the intact temporal lobe adjusts the internal information transmission state more rapidly. It may temporarily disengage connections with other regions that are less important, providing more support for the visual cortex to process visual movement information. Centrality of the intact temporal lobe demonstrates how much information is transferred through this area. In responders, we noticed a trend of local node enhancement in centrality and strength during FU compared with baseline and control. In responders, the centrality of Occipital_mid_IH remained unchanged compared to baseline, whereas centrality of Temporal_mid_IH increased. Considering the global coherence enhancement between the intact occipital and intact temporal regions, this suggests that the communication between the intact occipital and temporal lobe plays a critical role in visual function enhancement, and it is this enhancement that seems to be adaptive.

In summary, occipital damage following stroke creates partial vision loss with brain network alteration in the delta and alpha band, and ACDC, but not AC alone, improves visual functions. As we now showed, brain network plasticity patterns such as interhemispheric (im-) balance and long-lasting plasticity were consistent with behavior performance in the ACDC group. This demonstrates that modulating brain network plasticity is a promising tool to induce vision recovery. An analysis of responders vs. non-responders (irrespective of the treatment they received) also helped us understand NIBS effects, highlighting the role of a reduction of the coherence between the LH and IH occipital lobes in the delta band and a reduced high alpha-band coherence between the frontal and occipital lobe; these two FCN patterns might comprise biomarkers of vision recovery and shed light on the role of coherence between intact occipital and intact temporal regions. Future experiments are needed to confirm this proposal.

### Limitations

Our study has several limitations. First, the sample size per group was relatively small, but recruitment problems to find patients who met all inclusion and exclusion criteria were a challenge. While finding significant effects despite a small sample is suggestive, our results are not conclusive until a larger-sample study is done. Another limitation is the low spatial resolution of the EEG signals and a common fMRI template from MNI when resourcing. A better resolution would be preferable with individual head model for both patients and controls. Some researchers reported that intraindividual variability in response to tDCS and tACS was found (90–93). There are different possible sources of outcome variability. First, the individual anatomy varies between patients, which generates differences in electric fields inside the brain (91). Even in a fixed stimulation montage and intensity, there is substantial variability of spatial distribution and strength considering individual anatomical differences (91). In our study, the data were warped into MNI space during source reconstruction. Some researchers argue that the standard template would make the anatomy less precise especially in the lesion area; however, because we used the same pipeline and could still demonstrate consistent and interpretable results from our brain network analysis, this limitation is acceptable for an exploratory study. Second, there may be other systemic factors (such as blood flow differences, hormonal or nutritional influences) that may influence neurophysiological activity during FU, and third, we recently observed that vision recovery following ACS treatment in patients with optic nerve damage depends on patients' personality traits and stress history (94).

## Conclusions

Our exploratory clinical trial of hemianopic stroke patients showed that ACDC, but not ACS treatment, is able to induce greater hemispheric balance of brain FCNs in the alpha band, which correlates with vision recovery. In addition, ACDC decreases delta band coherence between the lesioned and intact occipital cortex and modifies the connections with other regions. A lowered global clustering coefficient observed in responders may be a physiological biomarker or mechanism of vision recovery, in that the brain's FCN can process visual information more efficiently. Here, visual processing is achieved with lower functional connectivity in the alpha band. In summary, brain FCN reorganization is relevant for the postlesion response and plasticity of the damaged visual system. This finding can inspire our search for more effective stimulation protocols to induce vision restoration in ways that are more effective and more long-lasting.

## Data Availability Statement

The raw data supporting the conclusions of this article will be made available by the authors, without undue reservation.

## Ethics Statement

The studies involving human participants were reviewed and approved by Ethics Committee of University Magdeburg. The patients/participants provided their written informed consent to participate in this study.

## Author Contributions

JX: design of the analysis and interpretation, statistics, and drafting and revision of the manuscript for intellectual content. ZW and AN: critical revision of the manuscript for intellectual content. BS: design and conceptualized study, interpretation of the data, and drafting and revision of the manuscript for intellectual content. All authors contributed to the article and approved the submitted version.

## Funding

The study was funded by the German Federal Education and Research Ministry, grant ERA-net Neuron (BMBF 01EW1210) to BS “REVIS” (Restoration of Vision after Stroke), the Otto-von-Guericke-University of Magdeburg and the Chinese Scholarship Council (stipend to JX). The sponsors had no involvement in the study design, the collection, analysis and interpretation of data, drafting the report; nor in the decision to submit the article for publication.

## Conflict of Interest

BS is shareholder of an out-patient clinical service which offers NIBS and NIBS devices. The remaining authors declare that the research was conducted in the absence of any commercial or financial relationships that could be construed as a potential conflict of interest.

## Publisher's Note

All claims expressed in this article are solely those of the authors and do not necessarily represent those of their affiliated organizations, or those of the publisher, the editors and the reviewers. Any product that may be evaluated in this article, or claim that may be made by its manufacturer, is not guaranteed or endorsed by the publisher.
